# Examination of Sex-Specific Participant Inclusion in Exercise Physiology Endothelial Function Research: A Systematic Review

**DOI:** 10.3389/fspor.2022.860356

**Published:** 2022-03-25

**Authors:** Lindsay A. Lew, Jennifer S. Williams, Jenna C. Stone, Alicia K. W. Au, Kyra E. Pyke, Maureen J. MacDonald

**Affiliations:** ^1^Cardiovascular Stress Response Lab, School of Kinesiology and Health Studies, Queen's University, Kingston, ON, Canada; ^2^Vascular Dynamics Lab, Department of Kinesiology, McMaster University, Hamilton, ON, Canada

**Keywords:** vascular function, endothelial function, sex-inclusion, sex-bias, exercise, flow-mediated dilation

## Abstract

**Background:**

To combat historical underrepresentation of female participants in research, guidelines have been established to motivate equal participation by both sexes. However, the pervasiveness of female exclusion has not been examined in vascular exercise physiology research. The purpose of this study was to systematically quantify the sex-specific prevalence of human participants and identify the rationales for sex-specific inclusion/exclusion in research examining the impact of exercise on vascular endothelial function.

**Methods:**

A systematic search was conducted examining exercise/physical activity and vascular endothelial function, assessed via flow mediated dilation. Studies were categorized by sex: male-only, female-only, or mixed sex, including examination of the sample size of males and females. Analysis was performed examining sex-inclusion criteria in study design and reporting and rationale for inclusion/exclusion of participants on the basis of sex. Changes in proportion of female participants included in studies were examined over time in 5 year cohorts.

**Results:**

A total of 514 studies were identified, spanning 26 years (1996–2021). Of the total participants, 64% were male and 36% were female, and a male bias was identified (32% male-only vs. 12% female-only studies). Proportions of female participants in studies remained relatively constant in the last 20 years. Male-only studies were less likely to report sex in the title compared to female-only studies (27 vs. 78%, *p* < 0.001), report sex in the abstract (72 vs. 98%, *p* < 0.001) and justify exclusion on the basis of sex (15 vs. 55%, *p* < 0.001). Further, male-only studies were more likely to be conducted in healthy populations compared to female-only studies (*p* = 0.002). Qualitative analysis of justifications identified four themes: sex-specific rationale or gap in the literature, exclusion of females based on the hormonal cycle or sex-differences, maintaining congruence with the male norm, and challenges with recruitment, retention and resources.

**Conclusions:**

This systematic review provides the first analysis of sex-based inclusion/exclusion and rationale for sex-based decisions in human vascular exercise physiology research. These findings contribute to identifying the impact of research guidelines regarding inclusion of males and females and the perceived barriers to designing studies with equal sex participation, in an effort to increase female representation in vascular exercise physiology research.

**Systematic Review Registration:**

CRD42022300388.

## Introduction

Sex-specific inclusion/exclusion in physiology research has been a long-standing issue, with male human, animal, and cell models often preferentially selected, as observed in basic science (Coiro and Pollak, 2019; Kim et al., 2021), pre-clinical human (Feldman et al., 2019), and clinical research trials (Heart and Stroke, 2018; Feldman et al., 2019). For example, a recent study by Cowley et al. examined sex-bias in sport and exercise science research, finding that during 2014–2020 in 6 major sports science journals, two-thirds of participants overall across studies were male and 31% of studies exclusively assessed males (compared to only 6% of studies exclusively assessing females) (Cowley et al., 2021). Further, these authors consistently observed sex-bias in the number of participants and number of sex-specific studies over the years of study (Cowley et al., 2021), which agreed with earlier findings by Costello et al., of studies published between the years of 2011 and 2013 (Costello et al., 2014). However, information is lacking on the rationale(s) for inclusion/exclusion on the basis of sex, or additional elements of sex-bias in the presentation of the articles examined, such as how information on sex is reported in the abstract and methodology of the manuscripts (Wilson et al., 2020).

“Sex” refers to the biological attributes, such as chromosomes, anatomy, and hormones, which determine male and female sex, while “gender” refers to socially constructed identity, roles, and behaviors that govern men and women (Tannenbaum et al., 2016); however, a nuanced approach to sex/gender identifies these constructs as more complex than a binary categorization (Fausto-Sterling, 2012; Bhargava et al., 2021). Responding to the concerns regarding sex/gender representation in human research, expert guidelines, government policies, grant guidelines, and recent journal publication requirements have been established. For example, the Sex and Gender Equity in Research (SAGER) guidelines, established in 2012, detail how to consider sex/gender in research design and reporting (Heidari et al., 2016). Expanding to examine government policies, in Canada, three federally-funded research councils, established the “Tri-Council Policy Statement: Ethical Conduct for Research Involving Humans” (TCPS 2) first established in 2010, and updated in 2018 (Tri-Council Policy Statement, 2018). Article 4.2 of this statement identifies that “women shall not be inappropriately excluded from research solely on the basis of gender or sex,” recognizing the historical and discriminatory exclusion of women in human research (Tri-Council Policy Statement, 2018). Similarly, in the United States, originally dating back to 1994 but recently updated in 2017, all NIH-funded clinical research must consider sex/gender, alongside other participant characteristics such as race and/or ethnicity in study design “…to ensure that research findings can be generalizable to the entire population” (National Institute for Health Research, 2017). Likewise, in the United Kingdom in 2017, the NIHR-INCLUDE Framework and Guidance was established to provide a “roadmap” for improving inclusion and representation in health and care research, including examining under-served groups including groups based on sex (National Institute for Health Research, 2020; Witham et al., 2020). These recent guidelines from health research bodies have identified clear direction for sex-specific inclusive practices in research.

Further, recent changes in grant guidelines require researchers to consider sex and/or gender in establishing research studies for federal funding. These changes were recently quantified in a 10-year longitudinal study, evaluating integration of sex and/or gender in grant submissions to the Canadian Institutes of Health Research (CIHR) (Haverfield and Tannenbaum, 2021). This study found that integration of sex in grant submissions rose from 22% in 2011 to 83% in 2021; while integration of gender increased from 12 to 33% (Haverfield and Tannenbaum, 2021). Moreover, applications with high scores in the integration of sex/gender have a higher likelihood of being funded (sex: 92% higher, gender: 153% higher) (Haverfield and Tannenbaum, 2021). Finally, some journals have endeavored to create guidelines or requirements for justification of sex/gender inclusion in study designs. For example, the *American Journal of Physiology – Heart and Circulatory Physiology* recently released new requirements that as of January 2023, all studies must include both sexes/genders, unless there is “strong scientific justification” for studying a single sex (e.g., studying hormonal contraceptive use in females, studying prostate cancer in males) (Lindsey et al., 2021).

Despite the burgeoning body of literature and policy changes aimed at integrating sex/gender considerations in human research, females continue to be excluded. For example, a recent case study of Ontario's NSERC-funded programs on the inclusion of female participants in cardiovascular physiology research found that females were underrepresented in or excluded from 63% of studies, with no temporal changes since the establishment of the TCPS 2 policy in 2010 (Wilson et al., 2020). Further, the study interviewed a limited number of Principal Investigators with NSERC Discovery Grant funding and identified notions of a “male norm” contributing to the preferential selection of male research participants as males are seen as the “standard” research subject and the female body is seen as more complex with considerations regarding the menstrual cycle, technical difficulties in acquiring measures, and/or disease prevalence (Wilson et al., 2020). An example of this can be seen in a recent paper by Naylor et al., which examined comparisons in brachial and femoral artery function in male athletes and excluded females due to the potential influence of sex hormones on flow-mediated dilation (FMD) and the need for male-specific data (Naylor et al., 2021).

Macrovascular endothelial function is commonly assessed using a standard FMD test via vascular ultrasound technology, which examines the artery response to occlusion-induced hyperemia (Thijssen et al., 2019). The FMD response of the brachial artery is directly correlated with endothelial function of the coronary arteries (Raitakari and Celermajer, 2000), and endothelial function is of clinical relevance as its dysfunction is a precursor in the development of atherosclerosis, stroke, and hypertension (Yeboah et al., 2009). Current guidelines (updated in 2019) for the assessment of vascular endothelial function, and specifically FMD, detail that “premenopausal women should be examined in a standardized phase of the menstrual cycle, since hormonal changes can affect FMD” (Thijssen et al., 2019). However, recent studies from our lab groups have repeatedly identified lack of changes in FMD across the menstrual and oral contraceptive cycle (D'Urzo et al., 2018; Shenouda et al., 2018; Williams et al., 2019; Liu et al., 2021). In agreement, a recent meta-analysis found that the menstrual cycle has only a small effect on FMD, which was largely accounted for by methodological differences in FMD acquisition (Williams et al., 2020). Therefore, the topic of how to consider “controlling” for the hormonal cycle has been long debated, with a Point-Counterpoint discussion published in 2020 (Stanhewicz and Wong, 2020; Wenner and Stachenfeld, 2020a) and recent methodological guidance papers (Sims and Heather, 2018; Elliott-Sale et al., 2021). Ongoing discourse on the topic of hormonal cycling controls indicates that testing females during a standardized phase of the hormonal cycle (e.g., early follicular phase or placebo phase) is recommended (Thijssen et al., 2019); however, the need for control may depend on the study design and population of interest (Stanhewicz and Wong, 2020).

Despite the ongoing discourse surrounding the need to have more inclusion of female participants in exercise physiology research, quantification of the historical sex-specific inclusion in vascular exercise physiology studies and identification of rationale(s) for inclusion/exclusion has yet to be published. Therefore, the purpose of this study was to systematically quantify the sex-specific prevalence of participants and identify the rationale(s) for sex-specific inclusion/exclusion of participants in human research examining the impact of exercise on vascular endothelial function. FMD was selected as the primary outcome of interest for its clinical relevance and prevalence as a macrovascular assessment method. Aligned with previous studies identifying sex-bias in exercise physiology research (Costello et al., 2014; Cowley et al., 2021), we hypothesized observing a male sex-bias in vascular exercise research, with rationales for exclusion related to the perceived complexity of female bodies. However, we also anticipated that there would be significant improvements in sex parity in vascular exercise physiology research in recent years, in concert with the implementation of guidelines and policy addressing the issue.

## Methods

This systematic review was conducted following the Preferred Reporting Items for Systematic Review and Meta-Analysis (PRISMA) statement. This review was also registered with the International Prospective Register of Systematic Reviews (PROSPERO).

### Search Strategy

A systematic search was conducted to investigate the current and past prevalence of sex-specific participant inclusion in vascular exercise physiology research. Studies were selected for inclusion through a systematic search of three online databases EMBASE, MEDLINE, and SPORTDiscus, from inception to October 2021. The search strategy (Appendix A) was aimed to select articles evaluating macrovascular endothelial function in response to acute or chronic exercise or physical activity interventions, or cross-sectional studies examining athlete/active vs. non-athlete/sedentary populations. The search consisted of the following combination of keywords: “exercise” OR “training” OR “physical activity” or “athlete” OR “cycling” OR “running” AND “vascular function” OR “endothelial function” OR “endothelium-dependent dilation” OR “flow-mediated dilation” OR “flow mediated dilation.”

### Eligibility Criteria

Only peer-reviewed, original studies, written in English were eligible for inclusion in this review. Studies were excluded if they were not available in English, or were reviews (e.g., narrative, literature, systematic, meta-analyses), case studies, commentaries, letters to the editor, conference abstracts, or non-peer reviewed (e.g., thesis manuscripts). Studies must have included human participants (cell and animal models were excluded) of any age and clinical status (*Population*). Studies must have incorporated any type of exercise, training or physical activity intervention or a cross-sectional comparison (*Intervention*). Finally, studies were required to include flow-mediated dilation (FMD) methodology assessed via ultrasound technology as an outcome variable (*Outcome*).

### Study Selection

Eligibility of studies was assessed by two reviewers. Initial title and abstract screening for all studies was conducted independently by two reviewers (LAL and JSW). Any discrepancies about eligibility were settled through consensus following a discussion with the two reviewers (LAL and JSW). Next, a full-text screening was conducted independently by two reviewers (LAL and JSW). Similarly, any discrepancies about eligibility or the reason for exclusion were settled by consensus following a discussion with the two reviewers (LAL and JSW).

### Data Extraction

Data was extracted from each study by one of four reviewers (LAL, JSW, JCS or ACWA), following the piloting of the data extraction sheet (Appendix B). Information regarding participant and study characteristics, sex of participants, results, and discussion of sex/gender throughout the article were extracted from all included studies. Data extracted about participant and study characteristics included: age, hormonal status, clinical status, type of study, and exercise/physical activity intervention length and type. Data extracted about sex of participants included: sex of participants, total sample size, sample size of males and females, and questions regarding the reporting of sex throughout the manuscript (Appendix B). Data on studies confusing or conflating terminology for sex compared to gender (e.g., study examining biological males and females, but using the term gender, or interchanging with men/women), and whether studies examined gender were extracted. Finally, justification/rationale of inclusion/exclusion of sex throughout the manuscript was recorded, where applicable and available.

### Data Synthesis and Analysis

#### Quantitative Analysis

Quantitative data was aggregated and reported across all years as Chi squared analysis (Microsoft Excel 2016). Proportion of female participants included in studies was compared across cohorts of years (i.e., every 5 years) to examine changes over time in sex-specific inclusion in research trials, using one-way ANOVA with the factor being the year cohort. Games-Howell corrected *post-hoc* tests were conducted as the homogeneity of variance was violated (Levene's test, *p* < 0.001; SPSS, Version 22.0). Significance was set at *p* ≤ 0.05. Five-year cohorts were selected, as the range of studies included in the review spanned 25 and 5-year cohorts provides a reasonable number of groups for comparative analysis. The proportion of male-only, female-only and mixed-sex studies were examined. Mixed-sex studies were also assessed for proportion of females (40–60% proportion of females/total sample size = equal; < 40% females = unequal favoring males, > 60% females = unequal favoring females), as previously published (Wilson et al., 2020). Where a study did not specify sex (*n* = 11), an assumption was made that the study was in only males, as per Wilson et al. (2020). Number of studies including various participant characteristics, study designs and types of exercise interventions in each sex-specific inclusion grouping was examined and reported. Sex-based analysis performed in studies and discussion of sex/gender throughout the paper were examined and reported.

#### Qualitative Analysis

Qualitative data was thematically coded using reflexive thematic analysis to identify patterns and themes of rationales provided for sex-specific inclusion/exclusion (Braun and Clarke, 2021). Two authors (LAL and JSW) analyzed extracted quotes from the articles that provided justification or rationales for the inclusion/exclusion of either sex, and sorted related quotes and defined and named common themes for inclusion in the results.

## Results

### Study Selection and Characteristics

**Figure 1:**
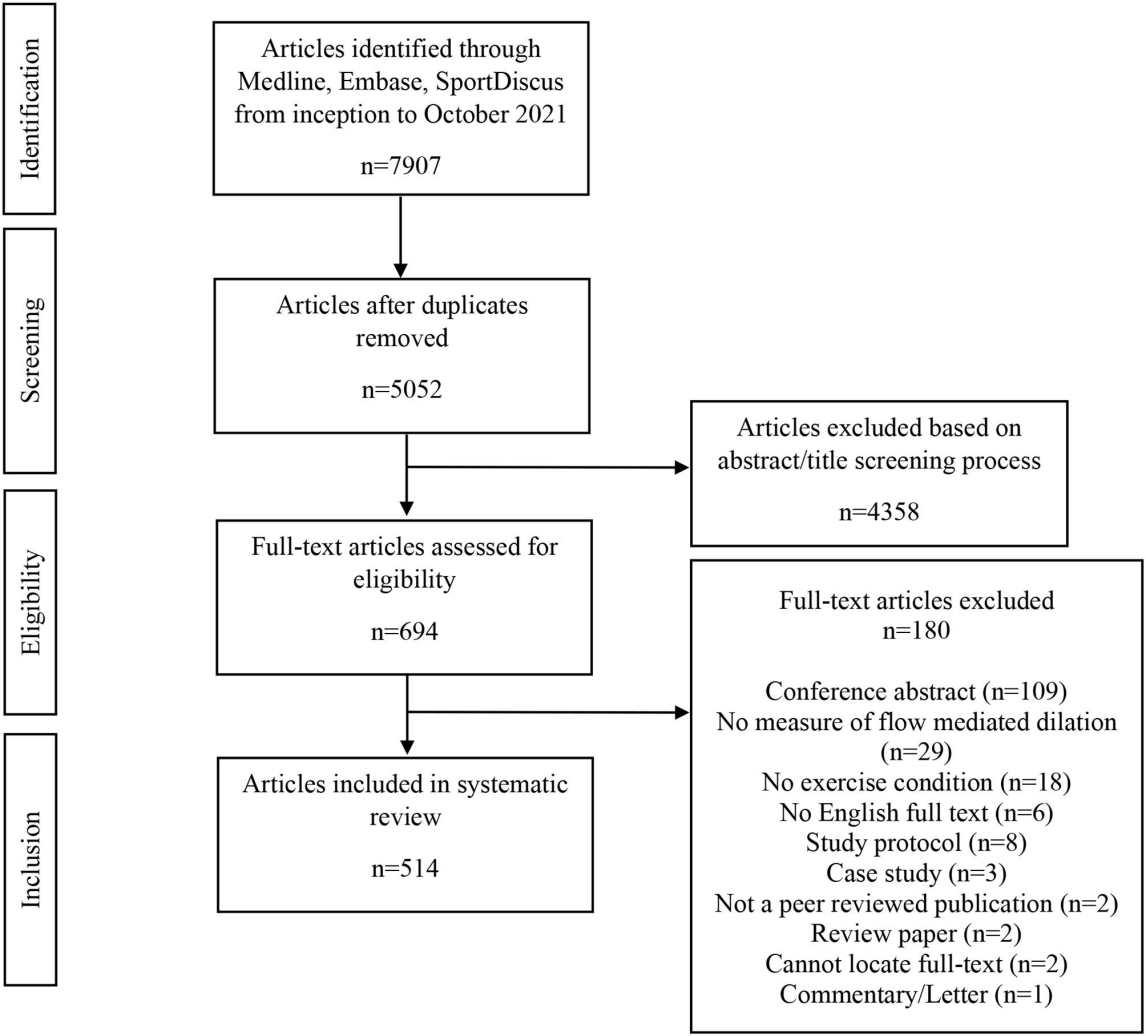
Systematic review flow-diagram.

**Figure 2:**
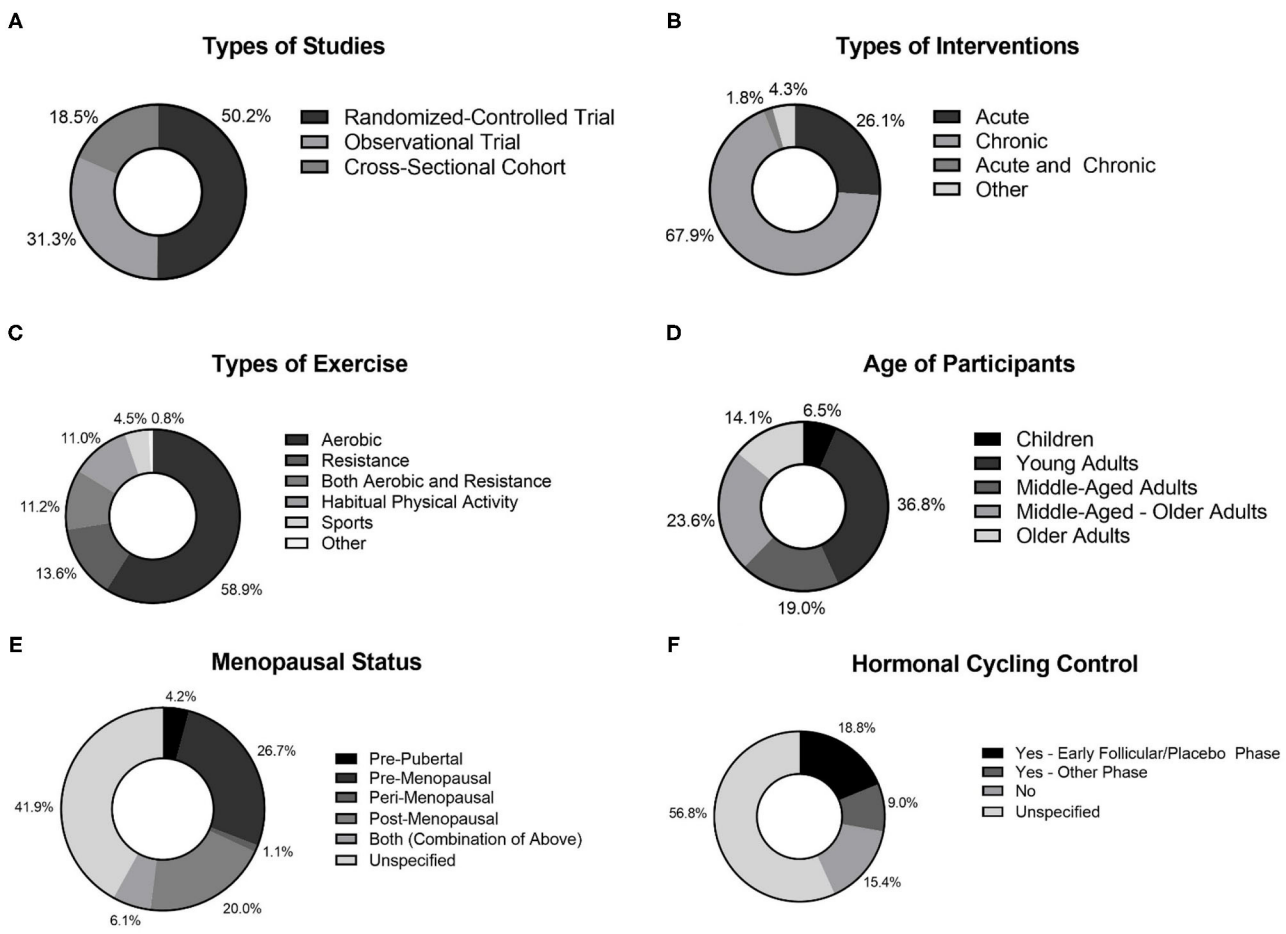
Study characteristics of studies included in review. **(A)** Type of studies; **(B)** Types of interventions; **(C)** Types of exercise; **(D)** Age of participants; **(E)** Menopausal status of female participants; **(F)** Hormonal cycling control in female participants.

The systematic search revealed 5,052 articles after duplicates were removed, that underwent title and abstract screening to result in 694 articles for full-text review. Following full-text review, 514 articles remained for inclusion in the systematic review, with exclusions identified in the flow diagram figure (Figure 1). Examining the year-ranges of studies, 37% (192) studies were published in 2021–2017, 30% (156) from 2016 to 2012, 21% (107) from 2007 to 2011, 10% (49) from 2002 to 2006, and 2% (10) from 1996 to 2001. Examining the types of participants included in the studies, there was an even split between healthy populations (49%) and clinical populations (51%). Further details regarding the types of studies included in this review can be found in Figure 2. The majority of trials were randomized controlled trials (Figure 2A), chronic exercise training interventions (Figure 2B), and specifically involved aerobic exercise interventions (Figure 2C). Similarly, the participants represented in the included trials varied by age (Figure 2D) and menopausal status (Figure 2E), with a large proportion of studies not reporting menopausal status (42%). Similarly, the majority of studies including female participants did not specify the phase of the hormonal cycle tested or did not control for the hormonal cycle (72%; Figure 2F); only 28% tested in a consistent hormonal phase (e.g., early follicular phase/placebo phase or another consistent phase) as per the FMD guidelines (Thijssen et al., 2019).

### Sex-Inclusion in Study Design

**Figure 3:**
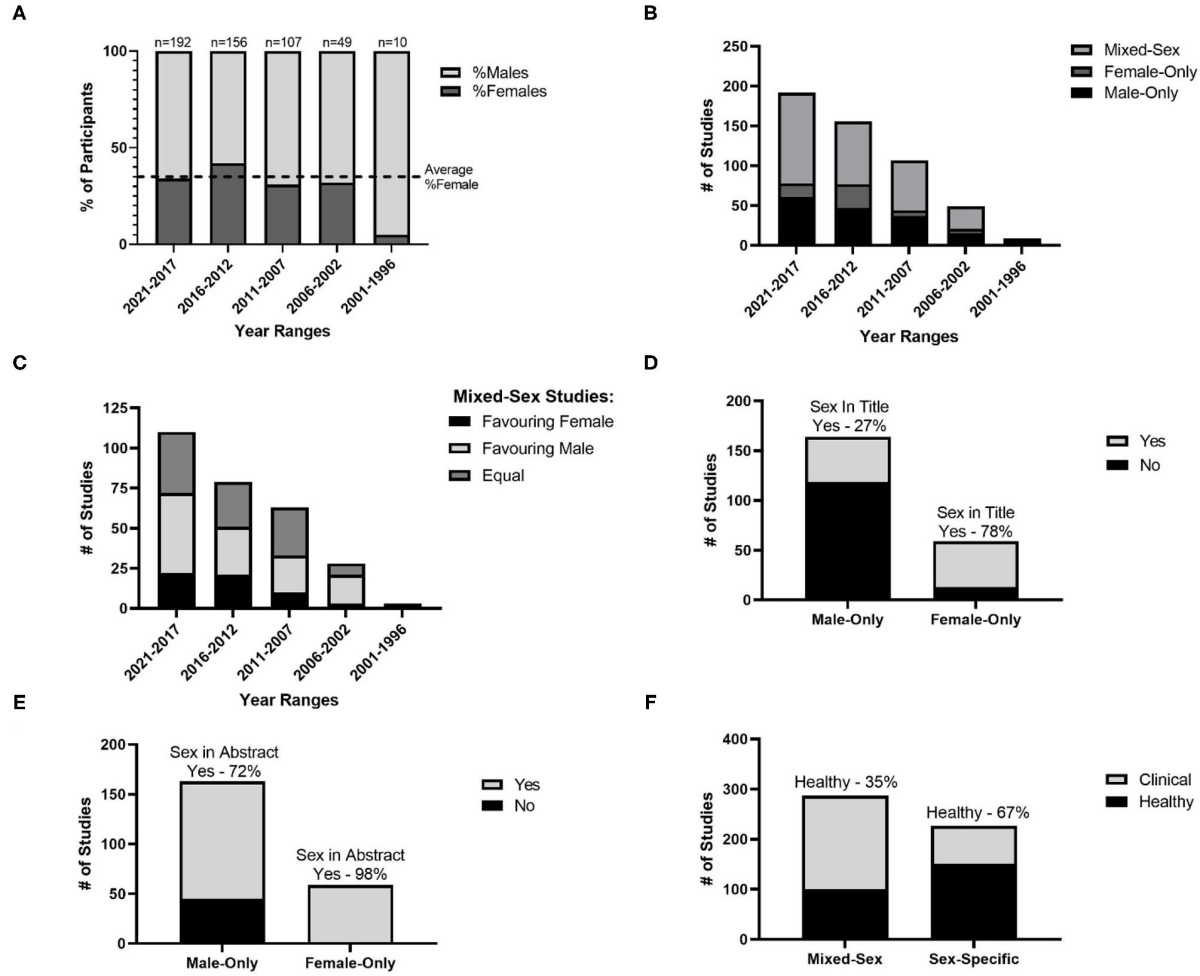
Sex-specific inclusion. **(A)** Average percentage of female and male participants from studies in cohorts of 5 years with the dotted line as the average over all studies; **(B)** Number of male-only, female-only and mixed-sex studies in cohorts of 5 years; **(C)** Number of mixed-sex studies with equal male-female participants, unequal participants favoring females and unequal participants favoring males, over cohorts of 5 years; **(D)** Number of male-only and female-only studies including sex in the article title; **(E)** Number of male-only and female-only studies including sex in the article abstract; **(F)** Number of studies in mixed-sex and sex-specific groups for healthy and clinical populations.

The total number of participants in the review was 25,364, with 16,140 males (64%) and 9,247 females (36%). The proportion of female participants in studies was different across time cohorts (main effect of time cohorts: *p* = 0.004); however, *post-hoc* testing revealed that the only difference was a lower %females in the 1996–2001 cohort compared to all others (*p* < 0.001). However, there were only 10 studies in this time cohort (2% of all studies). While the total number of studies increased, there was no difference in the proportion of female participants across time cohorts in the last 20 years (average: 35%; Figure 3A).

While the majority of trials reported sex of participants (97%), in 3% of studies sex was not disclosed; these studies were assumed to be “male-only” as described by Wilson et al. (2020). Examining further the number of female-only, male-only and mixed sex studies, the number of mixed-sex studies (56%) was greater than that of male-only studies (32%), which was greater than that of female-only studies (12%; Figure 3B). Of the studies that were mixed-sex, the number of studies that favored the inclusion of females (20%) was lower than the number of studies that included equal male and female participants (37%) and studies that favored inclusion of males (43%; Figure 3C).

### Sex-Inclusion in Study Reporting

Of studies in single-sex populations (i.e., male-only or female-only studies), ~40% of studies included sex in the title, while nearly 80% of studies included sex in the abstract. Similarly, of studies that were single-sex in nature or mixed-sex with an underrepresentation of one sex, ~17% of studies justified the exclusion or underrepresentation of a sex. Finally, 32% of studies in single-sex populations recognized the lack of generalizability of their study.

When comparing female-only and male-only studies, it was determined that male-only studies were less likely to report sex in the title compared to female-only studies [27% male-only vs. 78% female-only, χ^2^ (1,223) = 45.86, *p* < 0.001; Figure 3D]. The same was true for reporting sex in the abstract [72 vs. 98%, χ^2^ (1,222) = 17.71, *p* < 0.001; Figure 3E], and providing a justification for the exclusion on the basis of sex [15 vs. 55%, χ^2^ (1,206) = 29.20, *p* < 0.001]. However, there was no difference in the proportion of studies which identified sex-exclusion as a limitation in their ability to generalize from the study population [30% male-only vs. 39% female-only, χ^2^ (1,193) = 1.12, *p* = 0.291].

Further, when comparing mixed-sex and single-sex (i.e., male-only or female-only) studies, mixed-sex studies were more likely to be conducted in clinical populations (65% of mixed-sex studies versus 33% of single-sex studies), while single-sex studies were more likely to be conducted in healthy populations [35% of mixed-sex studies vs. 67% of single-sex studies; χ^2^ (1,514) = 50.90, *p* < 0.001; Figure 3F]. Examining whether male-only or female-only studies were driving this difference, it was determined that male-only studies were more likely to be conducted in healthy populations (72% of male-only studies vs. 50% of female-only studies), while female-only studies were more likely to be conducted in clinical populations [28% of male-only studies vs. 50% of female-only studies; χ^2^ (1,227) = 9.99, *p* = 0.002].

Finally, when examining the two most common exercise interventions in studies (i.e., resistance vs. aerobic), it was found that male-only studies were more likely to include resistance exercise interventions (26% of male-only studies vs. 15% of mixed-sex/female-only studies), while mixed-sex/female-only studies were more likely to involve aerobic exercise interventions [74% of male-only studies vs. 85% of mixed-sex/female-only studies; χ^2^ (1,373) = 7.50, *p* = 0.006]. In addition, male-only studies were more likely to include acute exercise interventions (44% of male-only studies vs. 20% of mixed-sex/female-only studies), while mixed-sex or female-only studies were more likely to be chronic exercise training studies [56% of male-ony studies vs. 80% of mixed-sex/female-only studies, χ^2^ (1,483) = 29.62, *p* < 0.001].

### Qualitative Analysis of Study Reporting

Examining the justifications provided for the exclusion or inclusion of certain sexes, there were four main themes: need to study a specific sex for a sex-specific rationale or a gap in the literature, the need to exclude females on the basis of the hormonal cycle, maintaining the male norm, and challenges with recruitment, retention and resources. One of the first justifications identified was the need to study a specific sex given the sex-specific nature of a condition or a clear gap in the literature. For example, studies highlighted common sex-specific conditions in females, such as menopause or the influence of hormone therapy, polycystic ovarian syndrome, pregnancy and amenorrhea, or in males, such as prostate cancer and testosterone therapy. Similarly, the decision to only examine one sex was reported in some studies to be based on a paucity of literature in that sex.

**Figure 4:**
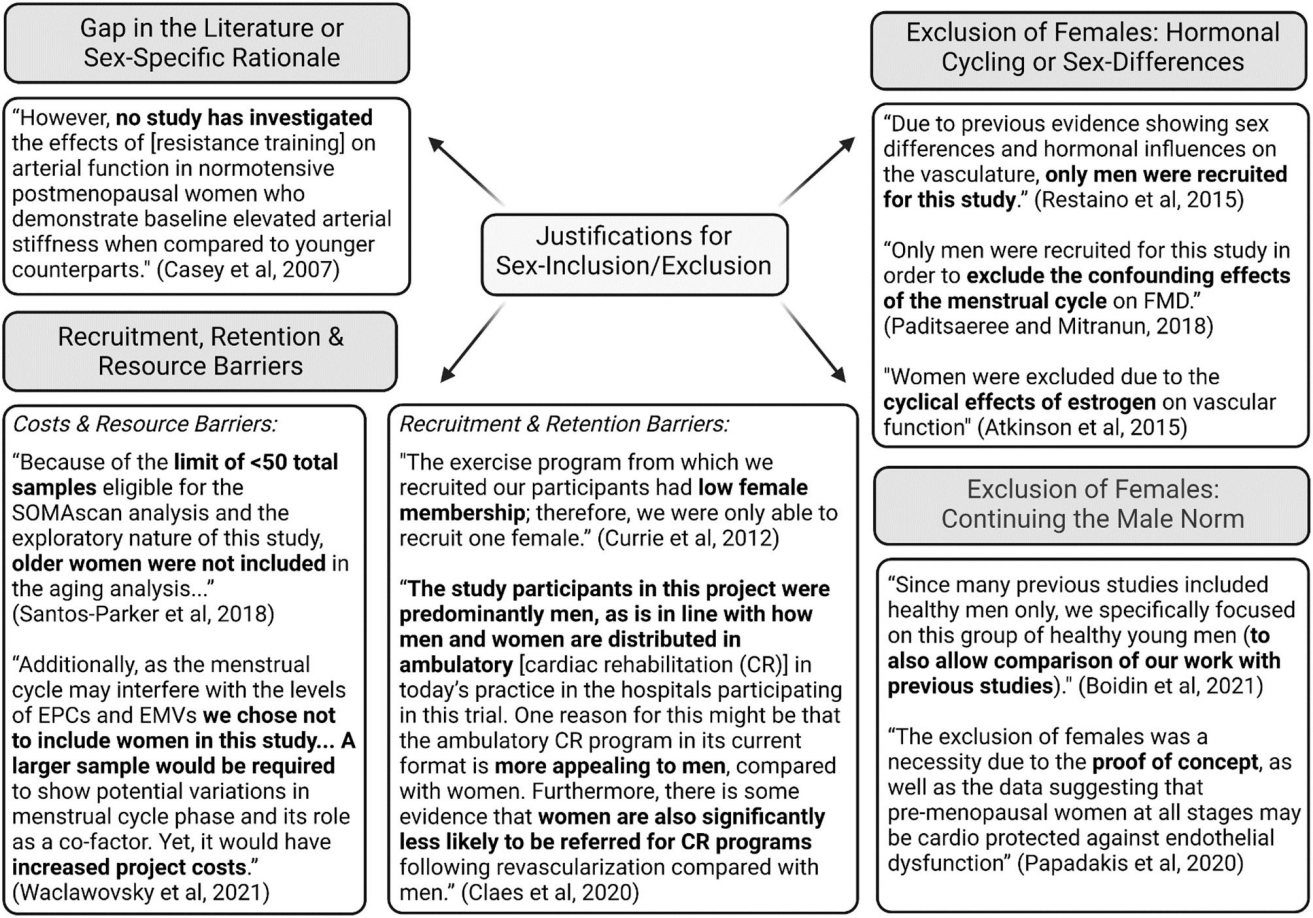
Summary of qualitative themes for sex-inclusion/exclusion and representative quotes.

Another common theme in the studies was exclusion of female participants on the basis of the hormonal cycle and/or attempting to remove the influence of sex that may confound the study findings. Alongside this theme was the notion of pursuing research that aligns with past populations as a “proof of concept,” aligning with past identification of a male norm. An additional recurring theme in the studies was the notion that researchers may face recruitment, retention and resource barriers when attempting to recruit both sexes. For example, financial barriers were identified, stating that it is more costly to examine sex equally. Several other studies identified challenges with recruitment and retention, citing low numbers of females as part of exercise programs or where clinical conditions are more common in males compared to females. Examples of these themes are illustrated in quotes in Figure 4 (Casey et al., 2007; Currie et al., 2012; Atkinson et al., 2015; Restaino et al., 2015; Paditsaeree and Mitranun, 2018; Santos-Parker et al., 2018; Claes et al., 2020; Papadakis et al., 2020; Boidin et al., 2021; Waclawovsky et al., 2021). In examining the identification of where generalizability is limited, studies primarily highlighted that because of the sex-specific nature of the study or the lack of equal participants across sex, study findings could not be generalized to other populations.

### Sex-Differences

Within studies examining mixed-sex populations, only ~10% of studies intended to test for sex-differences in their methods with an *a priori* design, with approximately one-third of these studies disaggregating based on sex to perform the analysis, and two-thirds of these studies incorporating sex-based comparison into their statistical analysis. Similarly, when considering all mixed-sex studies, only 17% reported analyzing data based on sex and reporting differences or lack thereof. Of studies reporting on sex-differences, 83% reported no sex-differences, while 17% reported that there were sex-differences in response to an exercise-based intervention. Finally, examining all studies in the review, only 20% included a discussion on how sex/gender may or may not influence the study's results; in mixed-sex studies, only 22% included a discussion on sex/gender.

### Examining Gender

Examining the inclusion of gender in the studies, approximately 40% of studies conflated or confused the terms sex and gender in reporting on participants (e.g., using terms “male” and “female” as gender instead of sex or using terms “woman” and “man” as sex instead of gender, or using both the terms “sex” and “gender” interchangeably throughout the paper). In addition, the vast majority of studies did not explicitly examine gender (e.g., identity, roles, behaviors) and its impact on the outcome (i.e., FMD) (99%).

## Discussion

### Summary of Study Findings

The purpose of this study was to quantify and characterize sex-specific prevalence of human participants in research examining the impact of exercise on vascular endothelial function and to identify the rationales justifying sex-specific inclusion/exclusion of participants. Overall, it is evident that females remain underrepresented in vascular exercise physiology studies, as indicated by a lower total prevalence of both female participants and female-only studies. Summarizing over 500 studies with ~25,000 participants, this study found evidence of a male-bias, with male participants included more than female participants (64 vs. 36%), and 32% of studies conducted with male-only populations (compared to 12% in female-only populations). In mixed-sex studies, favoring of female participants was less common (20%) than favoring of male participants (43%) or equal male and female representation (37%). Furthermore, underrepresentation of female participants was largely unaltered across time despite the advances in policy and recommendations related to sex and gender considerations. In addition, this study found that male-only studies were less likely to report sex in the title and abstract, and justify exclusion on the basis of sex, compared to female-only studies. Further, our analysis found that male-only studies tended to be conducted in healthy populations and involve acute interventions and resistance exercise interventions. Qualitative analysis found common rationales regarding unequal sex inclusion to be based on sex-specific conditions or paucity of research in a given sex, female exclusion on the basis of the hormonal cycle or sex-differences, perpetuation of the male norm, and concerns regarding the recruitment, retention and resources needed to pursue sex-parity. Finally, only 17% of mixed-sex studies performed sex-based analysis, demonstrating the paucity of sex difference research in the field of vascular exercise physiology, even in those studies that included mixed-sex in their participant pools.

### Sex-Specific Inclusion

Approximately one-third (36%) of participants included in all studies were female, highlighting the imbalance between male and female participant inclusion in vascular endothelial exercise physiology research. Although this present study was narrow in scope including only studies assessing FMD in response to an exercise intervention, the results align with similar research of broader scope (Costello et al., 2014; Wilson et al., 2020; Cowley et al., 2021). A case study of research by five cardiovascular physiology investigators in Ontario reported a slightly lower average female enrollment of 24% (Wilson et al., 2020). Moreover, two studies investigating the sex of participants in original articles of three (Costello et al., 2014) and six (Cowley et al., 2021) high-impact sport and exercise medicine journals similarly found that females participants encompassed 39 and 34% of all included participants, respectively. The proportion of single-sex studies further exemplifies the existence of a sex bias skewed toward male inclusion with 32% of the studies being male-only, and 12% female-only. This higher prevalence of male-only studies was also noted previously (Costello et al., 2014; Wilson et al., 2020; Woitowich et al., 2020; Cowley et al., 2021). For example, previous work in exercise physiology by Costello et al. identified 4–13% female-only proportion, Cowley et al. identified 6% female-only proportion, and work by Wilson et al. identified 5% proportion of female-only studies in vascular research (Costello et al., 2014; Wilson et al., 2020; Cowley et al., 2021). The lack of female-inclusion in research studies directly contributes to expanding gaps in basic biomedical and clinical understanding of how exercise influences vascular function in female cardiovascular systems. For example, with evidence of known sex-differences in vascular endothelial responses to exercise training (Seals et al., 2019), establishing sex-specific exercise training interventions is integral to improving cardiovascular health of both males and females and understanding underlying mechanisms responsible for sex-specific responses.

Interestingly, male-only studies are more likely to be conducted in healthy populations and utilize acute exercise interventions in comparison to female-only/mixed-sex studies. Alternatively, mixed-sex studies were more likely to be conducted in clinical populations compared to sex-specific studies. Discrepancies in prevalence of mixed-sex studies in clinical vs. healthy populations may stem from the specific policies and protocols mandated in clinical trials. For example, the National Institutes of Health (NIH) established the policy in 1994 for Inclusion of Women and Minorities to improve sex-based equality of participants in NIH-funded clinical research (NIH: Grants Funding, 2017). According to this policy, in addition to research proposals outlining female inclusion *a priori* and plans for appropriate outreach programs and activities to increase recruitment/retention of this population, investigators must also provide annual progress reports detailing sex/gender of participants. These mandatory checkpoints included in the rigorous clinical trial protocols increase the accountability of researchers to conduct mixed-sex studies. Whereas, equal inclusion of sex in participants is often recommended rather than mandated in research predominantly conducted in healthy populations and is not guided throughout the research process apart from investigator-driven design and funding body decisions. In addition, female-only inclusion may have been more prevalent in clinical studies as females are often studied for the complexity of sex-specific conditions and hormonal experiences (e.g., pregnancy, hormone use, menopause, amenhorrhea). Further, based on the observations in the qualitative findings of this review further detailed below in the *Reported Rationales for Exclusion* section, it may be speculated that females were disproportionately excluded from studies involving healthy populations and acute-based interventions, due to the perceived influence of hormonal cycle/sex hormones on basic mechanistic research outcomes.

### Reporting Sex in Studies

The SAGER guidelines were created in 2021 to promote a systematic reporting of sex and gender in research and provide greater transparency of scientific data (Heidari et al., 2016). Key components of these guidelines include reporting sex when detailing participant characteristics, as well as reporting the sex of participants in the title and abstract if only one sex is included. This present study provides evidence that many single-sex vascular exercise physiology studies fail to adhere to these guidelines as 3% did not detail sex, 60% did not report sex in the title, and 20% did not report sex in the abstract. Additionally, the SAGER guidelines emphasize the importance of reporting results disaggregated by sex and performing sex-based analysis when possible (Heidari et al., 2016). In this review, sex-based analysis was very limited, with only 10% of mixed-sex studies indicating an *a priori* decision to conduct sex-based analysis and 17% conducting a sex-based analysis. Alongslide the apparent need for more sex-difference research, there have been calls in the literature emphasizing the need for appropriately powered sex-based analysis (Aulakh and Anand, 2007).

Females undergo acute and chronic variations in sex hormones throughout their lives, including but not limited to cyclic fluctuations in endogenous sex hormones across the menstrual cycle or synthetic hormones across a contraceptive cycle, substantial reductions in sex hormones during the menopause transition, or temporary increases in synthetic hormones with the use of hormone therapy. As some evidence has suggested an impact of sex hormones on endothelial function (Hashimoto et al., 1995; Moreau et al., 2012) or modulating the endothelial response to exercise (Moreau et al., 2013), it remains imperative to report participant hormonal status. Specifically, identifying whether female participants are pre-menopausal, peri-menopausal, or post-menopausal, alongside details regarding hormonal cycle phase and contraceptive/hormone therapy use, where applicable, provides additional context for researchers to understand and interpret research findings.

Interestingly, reporting of hormonal status of female participants was limited in this review, with 42% of studies including females failing to specify hormonal status of participants. Further, there is little control for menstrual phase in studies including premenopausal women demonstrated by approximately three quarters of studies that did not control for menstrual phase or did not report phase. While controlling for hormonal cycle phase is debated (Stanhewicz and Wong, 2020), recent FMD guidelines suggest collecting data on premenopausal women in a standard phase of the menstrual cycle, alongside other standardized controls such as diet, exercise, alcohol/caffeine consumption (Thijssen et al., 2019). Considerations of the hormonal cycle should be evaluated similar to other necessary study design controls, considering the research question and study design, and establishing controls wherever possible. While there remains a need to *consider* the hormonal cycle in study design and reporting, female participants should not be excluded on the basis of hormonal variation, as female-inclusive research provides meaningful contributions to vascular exercise physiology. Altogether, these results suggest a need for not only improved quantity, but also quality, of reporting in vascular exercise physiology research conducted in females.

### Reported Rationales for Exclusion

In contrast to previous reviews (Costello et al., 2014; Cowley et al., 2021), this was the first study to examine justifications for exclusion on the basis of sex, and recognition of limits to generalizability with exclusion. This study found that male-only studies were less likely to provide justification for exclusion on the basis of sex, compared to female-only studies (15 vs. 55%), while both male-only and female-only studies equally recognized the limitations in generalizability of the study findings to broader population groups (30 vs. 39%). In examining the qualitative rationales for justification, four central themes emerged. First, sex-specific nature or a clear gap in the literature was a justified rationale for sex-exclusion, such as researchers exploring the influence of hormonal therapies like hormone replacement therapy or testosterone therapy, or sex-specific conditions like pregnancy and prostate cancer. The SAGER guidelines detail that sex-exclusion on the basis of a sex-specific research question is justified (Heidari et al., 2016). Similarly, some studies identified clear literature gaps in the introduction of the study, such as the recognition of the paucity of research in one sex for a given exercise intervention.

Another theme emerging from the qualitative analysis was the exclusion of females based on the more variable hormonal cycle influence, and the perceived need to perpetuate a “male norm” in aligning with prior research to ensure validity and comparison of study findings. This observation has been a consistent theme in the exclusion or underrepresentation of female participants for maintaining a status quo of studying males (Beery and Zucker, 2011; Yoon et al., 2014; Woitowich et al., 2020). This male bias has been identified in many fields, including both basic cell and animal research (Yoon et al., 2014) and human research, including physiology (Beery and Zucker, 2011; Will et al., 2017) and more recently in exercise physiology (Cowley et al., 2021). Further, qualitative examination of interviews by Wilson et al. identified that primary investigators believe this may be due, in part, to females being perceived as more complex, with considerations to the hormonal cycle (Wilson et al., 2020). Early work examining the influence of the menstrual cycle on vascular endothelial function established large fluctuations in FMD, and was used as a justification for excluding females on the basis of this complexity (Hashimoto et al., 1995). However, a recent meta-analysis has identified that the menstrual cycle may have only a small effect on vascular endothelial function, and variability may instead be explained by other methodological factors (Williams et al., 2020). Nonetheless, controlling for hormonal phase is still recognized as best practice, but should no longer be a justification for exclusion as several recent articles offer excellent guidance on how to account for hormonal status while investigating females (Sims and Heather, 2018; Wenner and Stachenfeld, 2020b; Elliott-Sale et al., 2021).

Finally, another theme that emerged from the qualitative analysis was various recruitment, retention and resource barriers that exist when attempting to recruit both sexes. For example, specifically in clinical studies, exclusion or underrepresentation of female participants was noted due to poor recruitment or higher prevalence of drop-outs. This is in line with earlier findings by Wilson et al., noting relative disease prevalence may limit recruitment efforts (Wilson et al., 2020). However, according to the SAGER guidelines, an effort to recruit equally across sex is necessary (Heidari et al., 2016), and concerted effort is required to target recruitment to participants who may be underrepresented due to inherent barriers in research participation. For example, women have been historically underrepresented in cardiac rehabilitation programs due to in part to systematic under referrals, and poorer retention due in part to other gender-based commitments such as family care and a lack of social support (Jackson et al., 2005; Supervía et al., 2017; Colbert et al., 2020). Finally, researchers noted as part of justification the concern that integrating male and female participants would result in increased costs for an experiment with the need to double sample size. While the increased costs associated with sample size and potential hormonal testing cannot be overlooked, it has been argued that some trials may not require an increased sample size when integrating both sexes (Beery, 2018), examining sex-specific responses in pre-clinical research may provide long-term savings at the stage of clinical trials, and further examining sex-differences may lead to future untapped areas of research development (Klein et al., 2015).

### Examining Gender

When reviewing study treatment of gender, approximately 40% of studies conflated or confused the terms sex and gender, often interchanging terminology throughout the article (e.g., using the term gender and then referring to “male” or “female” participants). In addition, almost no studies explicitly examined gender, specifically the socially constructed identity, behaviors, roles, and institutional interactions that humans experience, and that have been known to influence health (Tannenbaum et al., 2016). Within vascular exercise physiology research, exploring gender offers an untapped area of future research to offer a more nuanced approach to examining apparent sex-differences that cannot only be explained by biological sex variables, like chromosomes, anatomy, and hormones.

### Limitations

The scope of the project was narrowed to include only exercise physiology studies that utilized FMD to assess conduit artery endothelial function. Therefore, these results cannot be extrapolated further, although the findings of this project appear to be in line with studies of broader scope and in different disciplines (Costello et al., 2014; Wilson et al., 2020; Woitowich et al., 2020; Cowley et al., 2021). More research is needed to extend these findings to other assessments of cardiovascular function (e.g., arterial stiffness, carotid artery compliance, microvascular function) and other physiological interventions. Further, only English, peer-reviewed studies were included in this review. Thus, these findings do not encompass all vascular exercise physiology research due in part to the potential publishing bias against null or statistically insignificant results (Hopewell et al., 2009). Additionally, studies included in this review were from various geographic locations, however data was not extracted regarding the country in which the research originated. As such, differing local policies surrounding sex/gender inclusion in research and reporting, which may influence the integration of sex/gender (Merriman et al., 2021), could not be accounted for in the temporal analysis of sex-specific inclusion/exclusion. This remains an important future direction to understand the effectiveness of sex/gender guidelines and mandates from governing bodies. Similarly, journal-specific requirements for sex/gender reporting were not accounted for in the analysis, and this is therefore a potential future direction for research. Lastly, despite an attempt to mitigate investigator bias by creating a structured extraction template prior to analysis, the team of investigators are all female-identifying and share a common interest in integrating females into exercise physiology research which may have influenced the analysis and results.

### Guidance for Future Researchers

Based on the findings from this review, the first guidance for researchers is to consider improved data collection and reporting practices when considering sex/gender in future research studies. For example, sex identification in the title and abstract where studies are sex-specific. This recommendation can be reinforced by journals including reporting requirements around sex/gender during the manuscript submission and peer review process. For example, research by the *American Journal of Physiology – Heart and Circulatory Physiology* has found an increase in mixed-sex studies and reporting on sex/gender in articles since integrating strategies in manuscript submission and peer review (Lindsey et al., 2021). Similarly, work by Clayton and Tannenbaum have identified a simple structure of reporting of sex/gender in clinical research, noting that the *Journal of the American Medical Association* has integrated requirements for sex-specific reporting and justification for exclusion on the basis of sex (Clayton and Tannenbaum, 2016). However, journal instructions may not always result in improved sex/gender inclusion, and researchers should independently consider these factors in study design (Merriman et al., 2021). Further, reporting hormonal status of participants (e.g., menopausal status, hormonal cycling controls) is necessary to provide additional context to research findings. As detailed previously, researchers should consider how hormonal status, including hormonal cycling and hormonal therapy (including contraceptive use), may influence research outcomes and incorporate appropriate controls into research study design where appropriate; however, this should not come at the cost of arbitrary exclusion of female participants. Hormonal considerations should be balanced alongside other study design controls, with the central principle of inclusion of both males and females.

While it is recommended to aim for mixed-sex studies with equal male/female participation (Heidari et al., 2016), there are rationales for sex-specific research. For example, some thoughtfully designed studies included in this review identified the notion of “intentional design” for sex-specific research, where researchers noted a paucity of research or a direct rationale for sex-specificity (e.g., disease more prevalent in one sex, sex-specific condition), detailed in the introduction of a study. In contrast to the omission of rationale for sex-exclusion, or justification after design (e.g., in the discussion), it is recommended for researchers to consider sex-inclusion in the design of studies. However, as detailed in the review, some researchers have noted the limitations of sex-inclusion, specifically in clinical populations. For example, in some clinical populations recruitment and retention may be limited; however, researchers are still urged to work toward parity in study design and mitigate barriers for recruitment and retention on the basis of sex. Similarly, few studies examined sex-based analysis in study design; where appropriate, *a priori* analysis is encouraged to examine sex-differences in response to exercise interventions. Finally, some studies conflated sex/gender and nearly no studies examined more complex constructs within gender. Researchers are encouraged to consider including structured questions around sex/gender, such as the two-step sex and gender question (Bauer et al., 2017), or more in-depth gender questionnaires (Schmitt and Millard, 1988; Pelletier et al., 2015), alongside other sex/gender tools summarized in a recent review (McGregor et al., 2016).

## Conclusion and Future Directions

This is the first study to quantitatively assess sex-inclusion in vascular exercise physiology research studies over 25 years, building on prior research to qualitatively identify rationales for inclusion/exclusion on the basis of sex. There was clear evidence of the underrepresentation of female participants in vascular exercise physiology research, and this trend appears to be unaltered over time despite recent attention to this topic. In particular, healthy populations involving acute interventions appear to be an area for attention, recognizing increased rates of female exclusion in these studies. Researchers are urged to consider sex/gender inclusion in research study design and reporting, with the aim for improved female inclusion. With recent attention to the considerations of sex/gender in research study design, it is anticipated that future analyses in vascular exercise physiology research will identify improved sex-specific inclusion in the next 25 years.

## Data Availability Statement

The raw data supporting the conclusions of this article will be made available by the authors, without undue reservation.

## Author Contributions

JW and LL conceived and designed the study, with support from KP and MM. JW and LL performed searches, title and abstract screening, full-text screening, wrote the manuscript, and prepared figures. JW, LL, JS, and AA performed data extraction. All authors edited and revised the manuscript prior to final submission. All authors contributed to the article and approved the submitted version.

## Funding

JW, JS, AA, and MM are supported by a *Natural Sciences and Engineering Research Council* (NSERC) Discovery Grant to MM's Vascular Dynamics Lab (2019-05413). JW was supported by a *NSERC* Canadian Graduate Scholarship (Doctoral). LL was supported by an *NSERC* Postgraduate Scholarship (Doctoral). LL and KP are supported by an *NSERC* Discovery Grant to KP's Cardiovascular Stress Response Lab (2019-04894).

## Conflict of Interest

The authors declare that the research was conducted in the absence of any commercial or financial relationships that could be construed as a potential conflict of interest.

## Publisher's Note

All claims expressed in this article are solely those of the authors and do not necessarily represent those of their affiliated organizations, or those of the publisher, the editors and the reviewers. Any product that may be evaluated in this article, or claim that may be made by its manufacturer, is not guaranteed or endorsed by the publisher.
