# Supplementary material for: Examination of Sex-Specific Participant Inclusion in Exercise Physiology Endothelial Function Research: A Systematic Review
Source: Front Sports Act Living. 2022 Mar 25;4:860356. doi: 10.3389/fspor.2022.860356 (PMC8990239; doi:10.3389/fspor.2022.860356)
Supplement: Supplementary file 1 [file Data_Sheet_1.docx]

**Appendix A: Search Strategy**

The following search strategy was used for all three search engines:

1. exercise.mp
2. training.mp
3. physical activity.mp
4. running.mp
5. cycling.mp
6. athletes.mp
7. vascular function.mp
8. endothelial function.mp
9. endothelial-dependent dilation.mp
10. flow-mediated dilation.mp
11. flow mediated dilation.mp
12. 1 or 2 or 3 or 4 or 5 or 6
13. 7 or 8 or 9 or 10 or 11
14. 12 and 13

**Appendix B: Data Extraction Questions**

1. **Citation Name**
2. **Year of Study**
3. **Participant Characteristics**: Age (Based on Average Age)
   1. Children (<18)
   2. Young Adults (18-35)
   3. Middle Age Adults (35-55)
   4. Middle-Older Adults (55-65)
   5. Older Adults (65+)
4. **Participant Characteristics**: Hormonal Status, if testing females
   1. Pre-Pubertal
   2. Pre-Menopause
   3. Peri-Menopause (0-5 years Post-Menopause)
   4. Post-Menopause (5+ years Post-Menopause)
   5. Both
   6. Unspecified
   7. N/A (males only)
5. **Participant Characteristics**: If Pre-Menopause in #4, was phase controlled for?
   1. Yes – Early Follicular/Placebo Phase
   2. Yes – Other Phase
   3. No
   4. Unknown
   5. N/A
6. **Participant Characteristics**: Healthy/Clinical?
   1. Healthy
   2. Clinical
      1. If clinical, details (ex. cardiovascular, diabetes, smoking) – Text Box
7. **Study Design**: Primary Study Design
   1. Observational Trial
   2. Randomized Controlled Trial
   3. Cross-Sectional Cohort Trial
8. **Study Design**: Secondary Study Design
   1. Observational Trial
   2. Randomized Controlled Trial
   3. Cross-Sectional Cohort Trial
9. **Study Design**: Type of Exercise Intervention
   1. Acute
   2. Chronic
   3. Both acute and chronic
   4. Other
10. **Study Design**: Type of Exercise Intervention
    1. Aerobic
    2. Resistance
    3. Both Aerobic and Resistance
    4. Sport
    5. Habitual Physical Activity (Including Cross-Sectional)
    6. Other
11. **Study Design**: Additional Study Details (as needed) – Text Box
12. **Study Design**: Sex-Inclusion Study
    1. Female-Only
    2. Male-Only
    3. Mixed-Sex
    4. Sex Not Detailed
13. **Study Design**: Sample Size
    1. Total Sample Size
    2. # Males
    3. # Females
    4. If Mixed-Sex, % Females (# Females/Total Sample Size x 100%)
    5. If Mixed-Sex, Equal or Unequal Proportion
       1. 40-60% proportion female and male = equal
       2. Unequal favouring males (females lower than 40%)
       3. Unequal favouring females (males lower than 40%)
14. **Sex-Inclusion Questions:**
    1. Are the terms sex and gender conflated or confused in the study? (Yes, No)
    2. If only one sex is included in the study, is it evident in the title” (Yes, No, N/A)
    3. If only one sex is included in the study, it is evident in the abstract? (Yes, No, N/A)
    4. Is the study intended to test for sex-differences (i.e., an a priori hypothesis; power to determine)? (Yes, No, N/A)
       1. If yes, how: sex-disaggregation or sex-comparison?
    5. Is information about sex of participants available? (Yes, No)
    6. Is a justification provided for exclusion/underrepresentation of a particular sex? (Yes, No N/A)
    7. Reason to justify exclusion: Text box
    8. Does the study report and/or analyze data by sex? (i.e., reporting sex-differences or lack thereof) (Yes, No, N/A)
       1. If yes, was the difference across sex in endothelial function significant? (Yes – Both Sex, Yes – Females Only, Yes – Males Only, No – Both Sex, N/A)
    9. If single-sex, is limited generalizability based on the single-sex nature of the study acknowledged? (Yes, No, N/A – Mixed-Sex, N/A – Single-sex is inherent in the condition examined e.g., pregnancy, prostate cancer, contraceptive use)
       1. If yes, include details: Text box
    10. Is there a discussion of how sex/gender may or may not influence the study’s results? (Yes, No)
    11. Was gender examined (Yes/No)
        1. If yes, include details: Text box
